# Dual incorporation of non-canonical amino acids enables production of post-translationally modified selenoproteins

**DOI:** 10.3389/fmolb.2023.1096261

**Published:** 2023-01-24

**Authors:** Pearl Morosky, Cody Comyns, Lance G. A. Nunes, Christina Z. Chung, Peter R. Hoffmann, Dieter Söll, Oscar Vargas-Rodriguez, Natalie Krahn

**Affiliations:** ^1^ Department of Molecular Biophysics and Biochemistry, Yale University, New Haven, CT, United States; ^2^ Department of Cell and Molecular Biology, John A. Burns School of Medicine, University of Hawaii, Honolulu, HI, United States; ^3^ Department of Chemistry, Yale University, New Haven, CT, United States

**Keywords:** selenoproteins, acetyl-lysine, post-translational modifications, genetic code expansion, selenocysteine, synthetic biology

## Abstract

Post-translational modifications (PTMs) can occur on almost all amino acids in eukaryotes as a key mechanism for regulating protein function. The ability to study the role of these modifications in various biological processes requires techniques to modify proteins site-specifically. One strategy for this is genetic code expansion (GCE) in bacteria. The low frequency of post-translational modifications in bacteria makes it a preferred host to study whether the presence of a post-translational modification influences a protein’s function. Genetic code expansion employs orthogonal translation systems engineered to incorporate a modified amino acid at a designated protein position. Selenoproteins, proteins containing selenocysteine, are also known to be post-translationally modified. Selenoproteins have essential roles in oxidative stress, immune response, cell maintenance, and skeletal muscle regeneration. Their complicated biosynthesis mechanism has been a hurdle in our understanding of selenoprotein functions. As technologies for selenocysteine insertion have recently improved, we wanted to create a genetic system that would allow the study of post-translational modifications in selenoproteins. By combining genetic code expansion techniques and selenocysteine insertion technologies, we were able to recode stop codons for insertion of *N*_ε_-acetyl-l-lysine and selenocysteine, respectively, into multiple proteins. The specificity of these amino acids for their assigned position and the simplicity of reverting the modified amino acid *via* mutagenesis of the codon sequence demonstrates the capacity of this method to study selenoproteins and the role of their post-translational modifications. Moreover, the evidence that Sec insertion technology can be combined with genetic code expansion tools further expands the chemical biology applications.

## 1 Introduction

Selenium is an essential micronutrient required for the biosynthesis of selenocysteine (Sec), which is exclusively found in selenoproteins. Selenoproteins are involved in many key cellular functions, including calcium and redox homeostasis, cell maintenance, and immune and inflammatory responses (Ye et al., 2022). Twenty-five selenoproteins have been identified in humans, but their functions remain poorly characterized. This is partly due to the complicated and highly regulated Sec biosynthesis and its insertion into proteins. In eukaryotes, this process depends on *cis* and *trans-*acting factors in the mRNA and transportation of the tRNA to the ribosome, respectively (Pinkerton and Copeland, 2016). Similar to bacterial systems, the Sec-charged tRNA (Sec-tRNA^Sec^) recognizes a dedicated UGA non-sense codon with the assistance of the Sec-specific elongation factor (eEFSec). Redefinition of the UGA codon as Sec is mediated by a hairpin element known as the Sec insertion sequence (SECIS) found up to 1,600 nucleotides away from the UGA codon in the 3′-untranslated region (UTR) of eukaryotic selenoprotein mRNA. eEFSec brings Sec-tRNA^Sec^ to the ribosome in response to the SECIS element in a process that involves an additional factor, the SECIS binding protein 2 (SBP2) (Copeland et al., 2000). SBP2 is predicted to bind to the SECIS element, bending the 3′-UTR to interact with eEFSec, though the detailed mechanism is still not fully understood (Kossinova et al., 2014; Hilal et al., 2022).

The complicated translation path of eukaryotic selenoproteins poses a challenge to overexpress and purify these proteins for functional characterization studies. Some strategies [e.g., pSelExpress1, orthogonal aminoacyl-tRNA synthetase (aaRS):tRNA pairs] have been developed to address this challenge in eukaryotes [reviewed in (Novoselov et al., 2007; Chung and Krahn, 2022)]. However, the bacterial Sec insertion system is simpler, only requiring a specialized elongation factor (SelB) to recognize Sec-tRNA^Sec^ and the SECIS element for insertion of Sec. As a result, more methodologies have been developed for recombinant Sec insertion in bacteria [reviewed in (Chung and Krahn, 2022)]. We have specifically focused on engineering SECIS-independent translation by removing the requirement for SelB and instead using the canonical elongation factor (EF-Tu) (Aldag et al., 2013). This method depends on allo-tRNAs, a tRNA species with an unusual cloverleaf structure (Mukai et al., 2017). We have since verified that some of the allo-tRNAs are recognized by EF-Tu (Mukai et al., 2018) with tertiary structures that facilitates accommodation in the *E. coli* ribosome (Prabhakar et al., 2022). Through engineering strategies, they have been altered for efficient Sec incorporation (Mukai et al., 2018).

As with standard protein expression, eukaryotic selenoproteins can also be expressed in bacteria but with minimal post-translational modifications (PTMs). To install PTMs into *E. coli* expressed proteins, genetic code expansion (GCE) utilizes orthogonal aaRS:tRNA synthetase pairs and non-canonical amino acids (ncAAs) (Chen et al., 2018; Porter and Mehl, 2018; Yang et al., 2018). The aaRS is engineered to accept diverse ncAAs typically for insertion at a non-sense or stop codon. Some commonly used orthogonal translation systems include the *Methanocaldococcus jannaschii* tyrosyl-tRNA synthetase (TyrRS) and archaeal pyrrolysyl-tRNA synthetase (PylRS) systems. The tolerance of PylRS to anticodon changes in its cognate tRNA^Pyl^ makes it an attractive tool to recode any codon, while the codon choice for TyrRS can be more limiting due to its anticodon recognition (Crnković et al., 2016). These systems have been used to insert common PTMs such as acetylation (Lacoursiere et al., 2020), phosphorylation (Balasuriya et al., 2020), or both (Venkat et al., 2018).

The presence of three non-sense codons in the genetic code offers an opportunity to insert multiple ncAAs into a single protein by combining existing orthogonal systems. To this end, several studies have demonstrated the synthesis of proteins containing two and even three ncAAs (Neumann et al., 2010; Chatterjee et al., 2013; Wang et al., 2014; Dunkelmann et al., 2020; Tharp et al., 2021). Since tRNA^Sec^ is acylated with Ser by endogenous seryl-tRNA synthetase (SerRS) before conversion to Sec *via* dedicated enzymes, it may be compatible with many of these orthogonal translation systems. This was tested with the natural Sec machinery in *E. coli*. Using a natural eukaryotic selenoprotein with a penultimate Sec amino acid, mRNA engineering facilitated a SECIS element to be inserted into the 3′-UTR of the mRNA while still promoting recognition by endogenous SelB for Sec insertion. Combining this with AcKRS (an aaRS evolved from *Methanosarcinae* PylRS) for incorporation of *N*_ε_-acetyl-l-lysine (AcK) showed the compatibility of these two systems and their capability of introducing AcK into a natural selenoprotein (Wright et al., 2018). However, the requirement of a SECIS element immediately downstream of the UGA codon limits this strategy to natural bacterial selenoproteins or eukaryotic selenoproteins with a Sec residue at the C-terminal end (penultimate or ultimate state).

**FIGURE 1:**
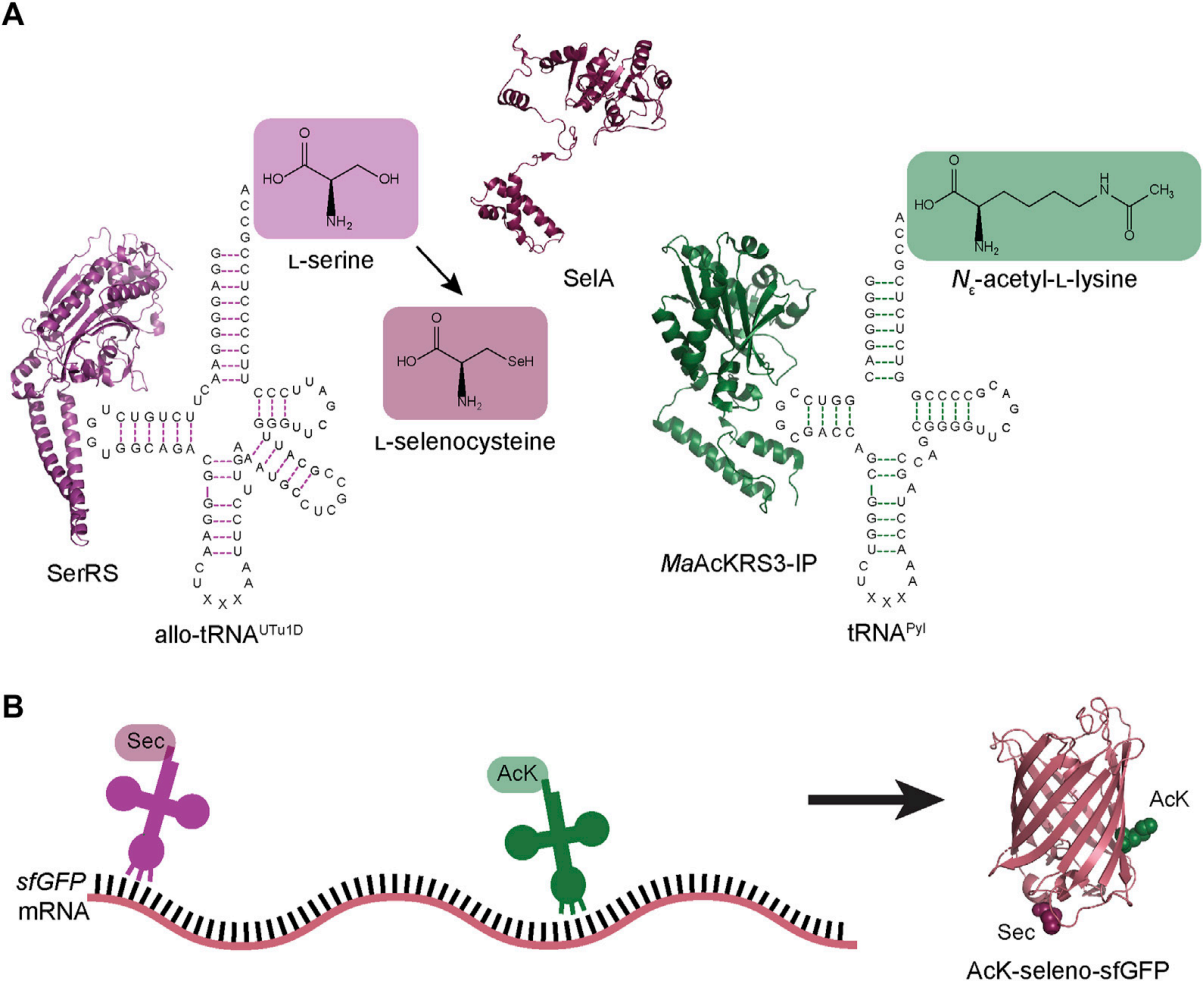
Schematic of dual ncAA incorporation to produce post-translationally modified seleno-sfGFP. **(A)** Endogenous *E. coli* seryl-tRNA synthetase (SerRS represented by PDB:1SER) aminoacylates allo-tRNA^UTu1D^ with l-serine, which is then converted to l-Sec by *A. salmonicida* selenocysteine synthase (SelA represented by PDB:3W1K). *M. alvus* PylRS containing mutations for recognition of *N*_ε_-acetyl-l-lysine (AcK) (*Ma*AcKRS3-IP represented by PDB:4Q6G) aminoacylates *M. alvus* tRNA^Pyl^. **(B)** Aminoacylated allo-tRNA^UTu1D^ and tRNA^Pyl^ insert Sec and AcK, respectively, at specific positions in the mRNA. These tRNAs are encoded to suppress stop codons (UAG or UGA), leading to the biosynthesis of AcK-seleno-sfGFP (represented by PDB:1EMB).

Here we present a new strategy for the simultaneous insertion of Sec and AcK into super-folder green fluorescent protein (sfGFP) and human glutathione peroxidase 1 (GPx1). We achieved this by combining a previously engineered Sec-incorporation system to site-specifically insert Sec anywhere in a polypeptide chain (Mukai et al., 2018) with a *M. alvus* PylRS engineered for insertion of AcK (Figure 1) (Seki et al., 2020). With the correct choice of anticodon for each of these translation systems, we facilitated increased codon orthogonality and suppression to yield post-translationally modified selenoproteins. This further expands the genetic code expansion toolbox, enabling the capability to design novel proteins and study eukaryotic selenoproteins in *E. coli*.

## 2 Materials and methods

### 2.1 General

Enzymes for molecular cloning were purchased from New England Biolabs and Takara Bio. Gene fragments were purchased from Integrated DNA Technologies and Twist Bioscience. W.M. Keck Foundation at Yale University provided oligonucleotide synthesis (Supplementary Table S1) and DNA plasmid (Supplementary Table S2) sequencing. Antibiotics and media additives were used at the following final concentrations: ampicillin (amp), 100 μg/mL; spectinomycin (spec), 50 μg/mL; glucose, 1% (w/v); arabinose, 0.1% (w/v); sodium selenite, 10 μM; *N*_ε_-acetyl-l-lysine (AcK), 10 mM; nicotinamide, 20 mM.

### 2.2 Plasmid construction

#### 2.2.1 pB_sfGFP_2TGA (pB_PM02) and pB_sfGFP_151TAG (pB_PM03)

The stop codon of the sfGFP gene in the previously reported pB_sfGFP plasmid (Chung et al., 2022) was mutated to TAA using the primer pair PM01/02 (pB_PM01). PM03/04 and PM05/06 primers were used separately to introduce a TGA at position 2 (2TGA) or a TAG at position 151 (151TAG) of sfGFP in the pB_PM01 plasmid. This provided the plasmids (pB_PM02 and pB_PM03) to test each suppression system individually.

#### 2.2.2 pB_sfGFP_2TGA-*Ma*AcLysRS3-tRNA^Pyl^_UCA_ (pB_PM06) and pB_sfGFP_151TAG-*Ma*AcLysRS3- tRNA^Pyl^_UCA_ (pB_PM07)

To insert the PylRS and tRNA^Pyl^ genes for AcK insertion into the previously designed plasmids, the primer pair PM07/08 was used to amplify the chemically synthesized *Ma*AcLysRS3-IP gene (Seki et al., 2020) and insert a proK promoter. Primers PM09/10 opened the pB_PM01, pB_PM02 or pB_PM03 plasmids downstream of the origin of replication with overhangs complementary to the amplified *Ma*AcLysRS3-IP fragment to insert in the reverse direction. The opened pB_PM01, pB_PM02 or pB_PM03 and *Ma*AcLysRS3-IP fragment were assembled with NEBuilder HiFi to generate pB_PM04, pB_PM05, and pB_PM06, respectively. These plasmids were then opened upstream of *Ma*AcLysRS3-IP with PM11/12 to insert the *M. alvus* tRNA^Pyl^_UCA_ fragment containing an Lpp promoter and rrcn terminator. The tRNA^Pyl^_UCA_ fragment was amplified with PM13/14 from a pBAD-sfGFP-tRNA plasmid (Tharp et al., 2020) containing the sequence for tRNA^Pyl^_UCA_. The opened pB_PM04, pB_PM05, and pB_PM06 plasmids and tRNA^Pyl^_UCA_ fragment were assembled with NEBuilder HiFi to generate pB_PM07, pB_PM08, and pB_PM09, respectively.

#### 2.2.3 pB_sfGFP_2TGA_151TAG-*Ma*AcLysRS3- tRNA^Pyl^_UCA_ (pB_PM10)

Starting with the pB_PM08 plasmid, PM05/06 primers were used to add the second stop codon within the coding region of sfGFP for dual suppression studies.

#### 2.2.4 pB_04_2TGA-*Ma*AcLysRS3- tRNA^Pyl^_UCA_ (pB_PM13)

The pB_04 plasmid containing sfGFP disrupted by the M86 DnaB mini-intein at position 204 was previously used to detect Sec insertion (Chung et al., 2022). The primer pair PM01/02 was used first to mutate the stop codon to TAA (pB_PM11). Due to the presence of a TAG already in the intein-sfGFP fusion, the second in-frame stop codon, TGA, was inserted at position 2 using the PM03/04 primers (pB_PM12). Insertion of the AcK-translation system (*Ma*AcLysRS3-mA17) was achieved as described in Section 2.2.2 (pB_PM13).

#### 2.2.5 pB_sfGFP-*Ma*AcLysRS3-tRNA^Pyl^_CUA_ plasmids (pB_PM14, pB_PM15, pB_PM16, and pB_PM17)

The anticodon sequence of the tRNA^Pyl^_UCA_ gene in plasmids pB_PM07, pB_PM08, pB_PM09, and pB_PM10 plasmids was mutated to CUA to suppress the UAG codon using the primer pair PM15/16. This generated plasmids pB_PM14, pB_PM15, pB_PM16, and pB_PM17, respectively.

#### 2.2.6 pB_04TGA_2TAG-*Ma*AcLysRS3-tRNA^Pyl^_CUA_ (pB_PM18)

The anticodon sequence of tRNA^Pyl^_UCA_ from pB_PM13 was also converted to CUA following the strategy described in 2.2.5. However, since the intein is specific for Sec insertion, the codons at position 2 of sfGFP and position 204 (position 1 of the intein) had to also be switched. Primer pairs PM17/18 and PM19/20 were used to convert 2TGA to 2TAG and pB_04 to pB_04TGA, respectively.

#### 2.2.7 pSecUGA

To recode UGA with Sec, the anticodon of allo-tRNA^UTu1D^ in the pSecUAG plasmid (Mukai et al., 2018; Chung et al., 2021) was changed to UCA. This was done using primer pair PM21/22 on the pSecUAG plasmid to generate pSecUGA.

#### 2.2.8 pB_GPx1_49TAG*-Ma*AcLysRS3-tRNA^Pyl^_CUA_ (pB_PM19)

For GPx1 expression, the human GPx1 gene was inserted into the pB*-Ma*AcLysRS3-tRNA^Pyl^_CUA_ vector containing genes for AcK insertion at UAG. Primer pair PM23/24 amplified GPx1_49TAG from pET-GPx1 (Mukai et al., 2018), while primer pair PM25/26 was used to open pB_PM17. Digestion with DpnI removed any parental plasmid from the PCR product before NEBuilder HiFi assembled the two PCR purified samples (Gpx1_49TAG and pB-*Ma*AcLysRS3-tRNA^Pyl^_CUA_). This produced plasmid pB_PM19.

#### 2.2.9 pB_GPx1_49TGA_114TAG*-Ma*AcLysRS3-tRNA^Pyl^_CUA_ (pB_PM21) and pB_GPx1_49TGA_148TAG-*Ma*AcLysRS3-tRNA^Pyl^_CUA_ plasmids (pB_PM22)

Mutagenesis of position 49 to encode a UGA was accomplished with primer pair PM27/28 on pB_PM19. This resulting plasmid (pB_PM20) was then subject to further mutagenesis to install UAG codons at position 114 or 148 (using primer pairs PM29/30 or PM31/32, respectively) for insertion of AcK. Final plasmids (pB_PM21 and pB_PM22) were then ready for dual insertion of Sec and AcK.

### 2.3 sfGFP fluorescence assay

Plasmids were transformed into electrocompetent *E. coli* cells (B-95.ΔA.Δ*fabR*Δ*selABC*) (Mukai et al., 2015) and plated on Luria Broth (LB) agar containing appropriate antibiotics and incubated overnight at 37°C. Single colonies were grown in 150 μL media at 37°C for 6 h in a 96-well black plate with clear bottoms. Unless otherwise noted, the media containing appropriate antibiotics included glucose, arabinose, sodium selenite, AcK, and nicotinamide was used. After 6 h, 75 μL from each well was transferred to a clean well in the same plate. The original wells were replenished with 75 μL of fresh media, while protein expression was induced in the new wells by adding 75 μL of media containing 2 mM isopropyl β-d-1-thiogalactopyranoside (IPTG). Fluorescent measurements and analysis were performed as described previously (Chung et al., 2022) using a minimum of four biological replicates.

### 2.4 Post-translationally modified selenoprotein production

For protein expression, pB_PM17, pB_PM21, or pB_PM22 plasmids were co-transformed with pSecUGA into electrocompetent *E. coli* cells (B-95.ΔA.Δ*fabR*Δ*selABC*) (Mukai et al., 2015) before plated on LB agar containing appropriate antibiotics and incubated overnight at 37°C. Single colonies were grown overnight at 37°C in 25 mL LB containing the appropriate antibiotics. Precultures were transferred to 1 L of LB containing antibiotics, arabinose, and sodium selenite and grown at 37°C. At an OD_600_ = 0.6, protein expression was induced with 0.5 mM IPTG, following the addition of nicotinamide, AcK, and extra sodium selenite. Proteins were expressed at 20°C for 16 h before pelleting the cells for purification.

All purification steps were performed under anaerobic conditions (90% N_2_, 5% H_2_, 5% CO_2_) in an anaerobic tent (Coy Laboratories). Each cell pellet was resuspended in 18 mL lysis buffer (50 mM sodium phosphate [pH 8.0], 300 mM NaCl, 30 mM imidazole, 10% (v/v) glycerol, 120 μg/mL lysozyme, 30 μg/mL Dnase, 0.5 mM phenylmethylsulfonyl fluoride [PMSF]) and 2 mL BugBuster^®^ 10 X Extraction Reagent (EMD Millipore). The lysed cells were incubated at room temperature for 20 min before the lysate was ultracentrifuged at 45,000 rpm (150,000 x g) for 45 min at 4°C. The supernatant was 0.45 μm filtered before being loaded onto a 1 mL Ni-NTA column pre-equilibrated with wash buffer (50 mM sodium phosphate [pH 8.0], 300 mM NaCl, 30 mM imidazole, 10% (v/v) glycerol). The beads were washed with 50 mL of wash buffer and then eluted in 1 mL fractions with elution buffer (50 mM sodium phosphate [pH 8.0], 300 mM NaCl, 250 mM imidazole, 10% (v/v) glycerol). Protein fractions were combined, concentrated, and buffer exchanged into storage buffer (50 mM sodium phosphate [pH 8.0], 300 mM NaCl, 10% (v/v) glycerol) before being stored at −80°C.

### 2.5 Mass spectrometry analysis

LC-MS analysis of dithiothreitol reduced samples were performed on a ThermoFisher Scientific Orbitrap Exploris 240 mass spectrometer, equipped with a heated electrospray ionization source (H-ESI) in positive ion mode with a ThermoFisher Ultimate 3000 RSLCnano HPLC System. On H-ESI source, sheath gas was set to 2 arbitrary units (arb), and auxiliary gas was set to 6 arb. The ion transfer tube was set at 275°C and the vaporizer temp at 200°C. The sample was analyzed on a MAbPac RP, 4 μM, 3.0 mm × 50 mm analytical column (ThermoFisher Scientific), held at 60°C. The protein was eluted at a rate of 500 μL/min for a 10-min gradient, where 0–7 min: 10%–70% acetonitrile + 0.1% formic acid; 7–8.2 min: 95% acetonitrile + 0.1% formic acid, 8.2–10 min: 20% acetonitrile + 0.1% formic acid. MS spectra were acquired using full scans at 15,000 resolution in the orbitrap within a range of 700–2,200 m/z. The maximum injection time was set at auto with a standard AGC target. Ten micro scans were employed, and the RF lens was set to 100%. 15 V of insource CID was applied. Thermo BioPharma Finder 5.1 was used for intact mass deconvolution and peak identification.

### 2.6 Western blot to identify acetylation

Approximately 5 μg of GPx1 protein samples were loaded onto a 4%–20% Mini-PROTEAN TGX stain-free gel (Bio-Rad) in reducing conditions. Samples were transferred to nitrocellulose and acetylation was detected with a primary mouse anti-acetylated lysine mAb (Novus Biologicals) and secondary anti-mouse IgG HRP-linked antibody (Cell Signaling Technology).

## 3 Results

### 3.1 Determination of suppression codon choice

**FIGURE 2:**
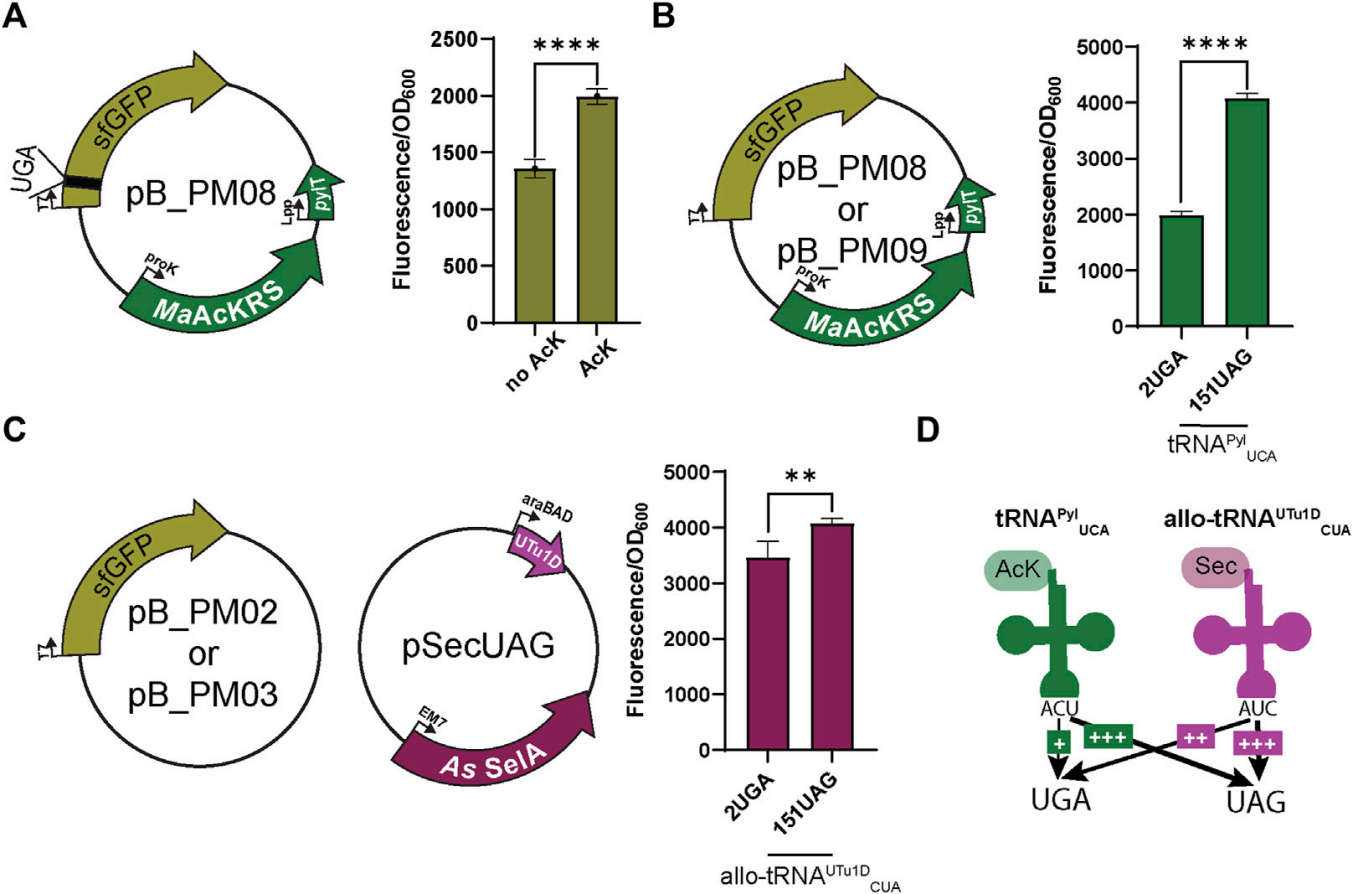
Fluorescence assay observes bias for UAG suppression. **(A)** To first test the specificity of the *Ma*AcKRS system for the presence of *N*_ε_-acetyl-l-lysine (AcK), pB_PM06 was used. This plasmid contains sfGFP under the control of a T7 promoter encoding a UGA at position 2 (2UGA), *Ma*AcKRS3-IP under a proK promoter, and tRNA^Pyl^_UCA_ (*pylT*) under an Lpp promoter. The presence of AcK promoted sfGFP fluorescence (*p* < 0.0001, *n* = 4). **(B)** pB_PM06 and pB_PM07 plasmids encoding 2UGA or 151UAG, respectively, were tested for the specificity of tRNA^Pyl^_UCA_ for its cognate codon (UGA). Significant increase in readthrough fluorescence (*p* < 0.0001, *n* = 4) was observed for 151UAG, the non-cognate codon. **(C)** Combining plasmids pB_PM02 or pB_PM03 encoding 2UGA or 151UAG, respectively, and pSecUAG allowed a test for UAG suppression by allo-tRNA^UTu1D^_CUA_. This plasmid contains the machinery for insertion of Sec, namely allo-tRNA^UTu1D^_CUA_ (UTu1D) under the araBAD promoter and *A. salmonicida* (*As*) SelA under EM7. Similar readthrough fluorescence was observed for both codons with a preference for its cognate codon, UAG (*p* < 0.01, *n* = 4). **(D)** Combining this information, tRNA^Pyl^_UCA_ (green) poorly suppresses its cognate codon UGA but is much better at suppressing UAG, while allo-tRNA^UTu1D^_CUA_ (magenta) also suppresses both codons with a bias towards UAG. Increase in suppression efficiency is denoted by an increase in + and bolder arrow.

Using our established pSecUAG plasmid system for insertion of Sec at UAG codons, we first engineered the anticodon of *M. alvus* tRNA^Pyl^ to recode UGA codons (tRNA^Pyl^_UCA_). Before testing the orthogonality of each tRNA for its respective codon, we confirmed the specificity of *Ma*AcLysRS3-IP for AcK (Seki et al., 2020). *E. coli* cells expressing only pB_PM08 showed significantly higher fluorescence (*p* < 0.0001) in the presence of AcK compared to cells without the amino acid (Figure 2A). This suggests that AcK promotes readthrough of the UGA codon in sfGFP with the *Ma*AcLysRS3:tRNA^Pyl^_UCA_ pair.

When using multiple translation systems to suppress more than one stop codon, confirming the codon specificity and orthogonality of engineered suppressor tRNAs is imperative. Therefore, we tested the ability of each system to readthrough UAG or UGA independently. To first investigate the ability of tRNA^Pyl^_UCA_ to specifically suppress UGA, we expressed the plasmid pB_PM08 or pB_PM09 in the presence of AcK. Notably, a significant increase (*p* < 0.0001) in GFP fluorescence for readthrough of 151UAG relative to 2UGA was observed (Figure 2B), suggesting that tRNA^Pyl^_UCA_ has a propensity to decode UAG codons better than its cognate UGA codon. We also tested this for allo-tRNA^UTu1D^_CUA_ by expressing plasmids pB_PM02 or pB_PM03 together with pSecUAG in the presence of sodium selenite. Interestingly, allo-tRNA^UTu1D^_CUA_ translated both 2UGA and 151UAG, with a significant increase in fluorescence (*p* < 0.01) observed for the intended UAG codon (Figure 2C). Together, these results show that tRNA^Pyl^_UCA_ and allo-tRNA^UTu1D^_CUA_ can suppress both UGA and UAG codons in isolation, displaying a higher efficiency to recode UAG (Figure 2D). The propensity to recode UAG was also observed when testing the suppression efficiency of each codon individually (pB_PM08 or pB_PM09) and together (pB_PM10) compared to wild-type (pB_PM07) in the presence of AcK and sodium selenite (Supplementary Figure S1A). Thus, these two tRNA variants are not entirely orthogonal.

**FIGURE 3:**
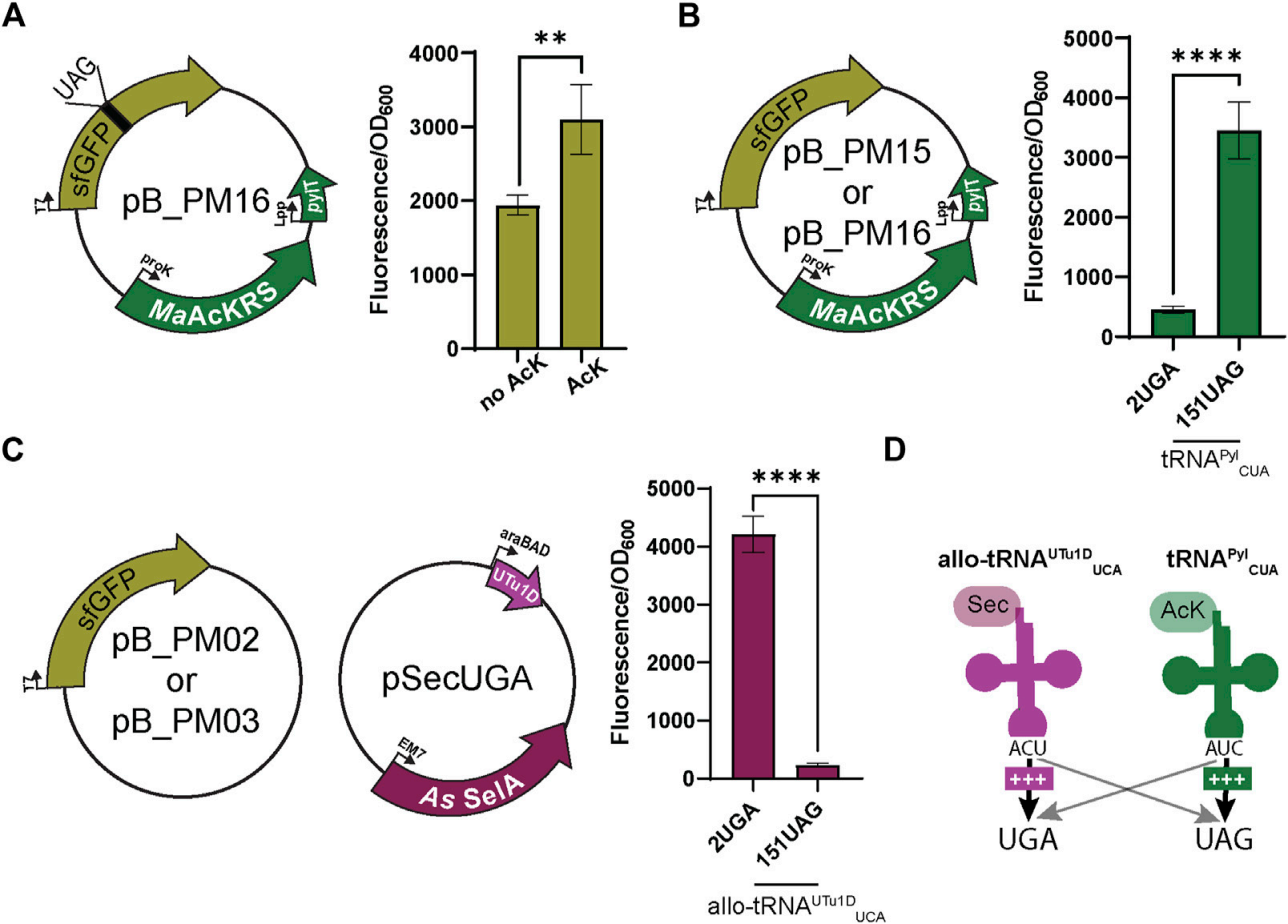
Anticodon swap creates codon orthogonality. **(A)** *N*_ε_-acetyl-l-lysine (AcK) was still found to be required for significant fluorescence (*p* < 0.0001, *n* = 4) of sfGFP with plasmid pB_PM13. This plasmid contains sfGFP under the control of a T7 promoter encoding a UAG at position 151 (151UAG), *Ma*AcKRS3-IP under a proK promoter, and tRNA^Pyl^_CUA_ (*pylT*) under an Lpp promoter. **(B)** pB_PM12 and pB_PM13 encoding 2UGA or 151UAG, respectively, were tested for the specificity of tRNA^Pyl^_CUA_ for its cognate codon (UAG). Significant increase in readthrough fluorescence (*p* < 0.0001, *n* = 4) was observed for 151UAG, the cognate codon, compared to 2UGA. **(C)** Combining plasmids pB_PM02 or pB_PM03 encoding 2UGA or 151UAG, respectively, and pSecUGA allowed a test for UGA suppression by allo-tRNA^UTu1D^_UCA_. This plasmid contains the machinery for insertion of Sec, namely allo-tRNA^UTu1D^_UCA_ (UTu1D) under the araBAD promoter and *Aeromonas salmonicida* (*As*) SelA under EM7. Significant increase in readthrough fluorescence (*p* < 0.0001, *n* = 4) was observed for 2UGA, the cognate codon, compared to 151UAG. **(D)** Combining this information, allo-tRNA^UTu1D^_UCA_ (magenta) is efficient at UGA suppression, while tRNA^Pyl^_CUA_ (green) is efficient at UAG suppression, creating an orthogonal system for dual suppression. Increase in suppression efficiency is denoted by an increase in + and bolder arrow. Grey arrows denote that no suppression was observed.

To test whether codon specificity can be attained, we swapped the anticodons of both tRNAs, resulting in tRNA^Pyl^_CUA_ and allo-tRNA^UTu1D^_UCA_. This led to the pB series (pB_PM14, pB_PM15, pB_PM16, and pB_PM17) and pSecUGA to decode UAG and UGA, respectively. We again confirmed that the *Ma*AcLysRS3:tRNA^Pyl^_CUA_ pair relies on AcK for efficient readthrough (*p* < 0.01) (Figure 3A). Importantly, we found that tRNA^Pyl^_CUA_ almost exclusively translates the UAG codon, with minimal decoding of UGA. Expressing only pB_PM15 or pB_PM16 we observed fluorescence when 151UAG is present but not in the presence of 2UGA (*p* < 0.0001) (Figure 3B). Similarly, allo-tRNA^UTu1D^_UCA_ displayed a higher specificity for UGA than UAG. Expressing pSecUGA with pB_PM02 or pB_PM03, we observed the opposite, fluorescence with 2UGA and not 151UAG (*p* < 0.0001) (Figure 3C). Furthermore, the suppression efficiencies of each codon individually (pB_PM15 or pB_PM16) and together (pB_PM17) were comparable and roughly 60%–70% of wild-type sfGFP (pB_PM14) in the presence of AcK and sodium selenite (Supplementary Figure S1B). These data suggest that allo-tRNA^UTu1D^_UCA_ and tRNA^Pyl^_CUA_ can simultaneously insert AcK at UAG and Sec at UGA into proteins efficiently (Figure 3D).

### 3.2 Dual insertion of AcK and Sec in a fluorescent reporter

**FIGURE 4:**
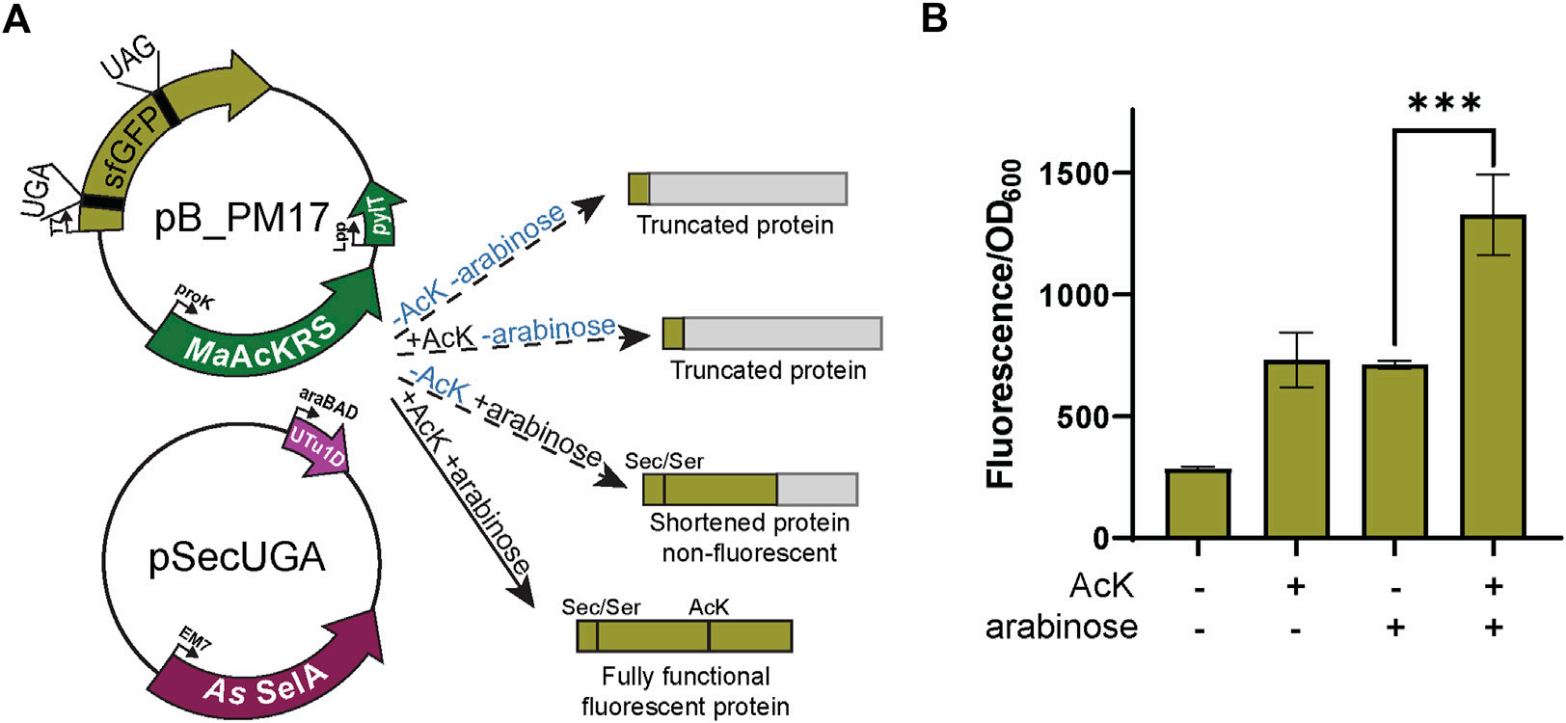
Fluorescence from dual suppression in sfGFP. **(A)** The assay was performed with a two-plasmid system: pB_PM14 and pSecUGA. The first plasmid contains the genes for *Ma*AcKRS3-IP and tRNA^Pyl^_CUA_ (*pylT*) for insertion of *N*_ε_-acetyl-l-lysine (AcK) at a UAG and pSecUGA contains the machinery for insertion of Sec at a UGA. In the presence of AcK, arabinose, and sodium selenite, the entire sfGFP gene should be expressed to produce fluorescence. In the absence of AcK or arabinose (or both), translation stops resulting in truncated non-fluorescent protein. Without arabinose, only the first amino acid would be translated. Without AcK, translation would continue until the UAG at position 151 to produce a truncated protein. **(B)** sfGFP assay shows successful readthrough of both codons in the presence of AcK and Sec with a significant increase in fluorescence (*p* < 0.001) compared to when only one or none of the components were added. Data are shown as the average of at least four biological replicates with the corresponding standard deviation shown with error bars.

**FIGURE 5:**
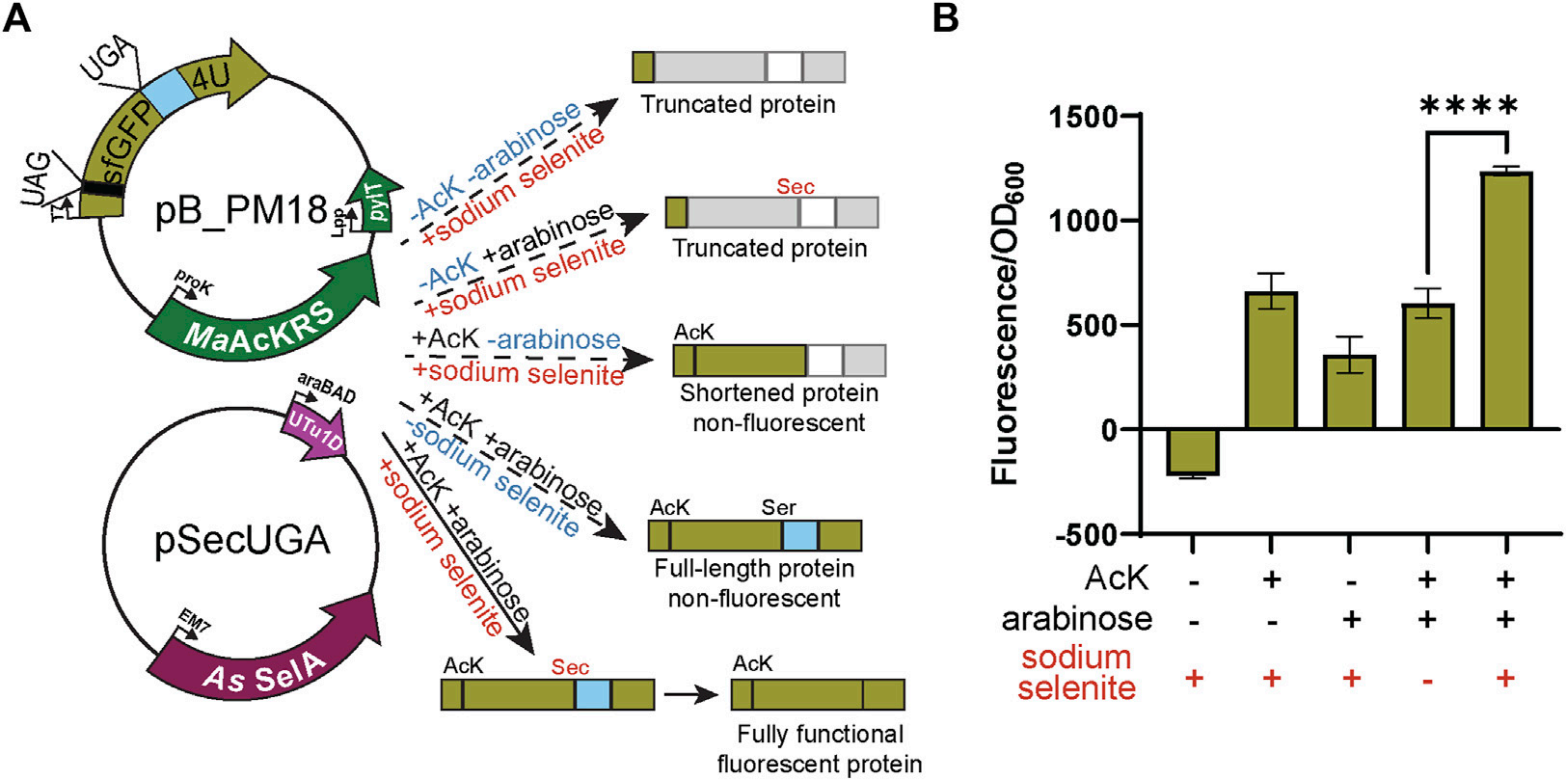
Confirmation of Sec insertion using intein reporter. **(A)** The assay was performed with a two-plasmid system: pB_PM15 and pSecUGA. The first plasmid contains the genes for *Ma*AcKRS3-IP and tRNA^Pyl^_CUA_ (*pylT*) for insertion of *N*_ε_-acetyl-l-lysine (AcK) at a UAG and a modified pB_04 sfGFP intein reporter gene. This gene has been modified from the original (Chung et al., 2022) to encode a UAG at position 2 and a UGA at position 204, the first position of the M86 mini-intein. The second plasmid, pSecUGA, contains machinery for Sec insertion at UGA. In the presence of AcK, arabinose, and sodium selenite, the entire sfGFP gene should be expressed. When Sec is inserted at position 204, the intein will splice out to produce a fluorescent functional reporter. In the absence of sodium selenite alone, the entire sfGFP gene should be expressed except serine (Ser) should be inserted at position 204, which does not induce splicing. This results in a non-functional, non-fluorescent protein. In the absence of AcK or arabinose (or both), translation stops resulting in truncated non-fluorescent protein. Only the first amino acid would be translated without AcK, while without arabinose, translation would stop at 204UGA. **(B)** The sfGFP_intein assay shows successful readthrough of position 2 and splicing of the intein in the presence of AcK, arabinose, and sodium selenite, with a significant increase in fluorescence (*p* < 0.0001) compared to when any one of the components is missing or in the absence of them all. Data are shown as the average of at least four biological replicates with the corresponding standard deviation shown with error bars.

Next, we combined the AcK and Sec incorporation systems to produce a synthetic post-translationally modified selenoprotein (AcK-seleno-sfGFP). We first tested the activities of the *Ma*AcLysRS3:tRNA^Pyl^_CUA_ and the Sec-translation systems (Figure 4A) in the presence and absence of AcK and arabinose (for allo-tRNA^UTu1D^_UCA_ expression). Significant sfGFP fluorescence (*p* < 0.001) was only observed in the presence of both AcK and arabinose but not if one or both is missing (Figure 4B), confirming the feasibility and compatibility of these two systems. Due to the possibility of Ser misincorporation by the pSecUGA system, we confirmed Sec incorporation using pB_PM18 (Figure 5A) which was engineered based on our previously developed Sec-specific sfGFP reporter (pB_04) (Chung et al., 2022). The sfGFP reporter is only functional (fluorescent) when Sec is inserted at amino acid 204 (position 1 of the M86 mini intein) to facilitate cleavage and splicing of the two sfGFP fragments (Appleby-Tagoe et al., 2011). Moreover, mutagenesis of codon 2 to UAG and the stop codon of the sfGFP to UAA allows for simultaneously monitoring the insertion of two ncAAs. By monitoring the fluorescence of the intein-sfGFP reporter, we only observed significant activity when both suppression systems and sodium selenite (Figure 5B) were present. These results corroborate that the fluorescence observed with the pB_PM17 plasmid (Figure 4B) is due to the presence of Sec. Moreover, the lack of fluorescence in the absence of AcK verifies its requirement and insertion at UAG (Figures 3A, 4B).

### 3.3 Expression of post-translationally modified seleno-sfGFP in *E. coli*

**FIGURE 6:**
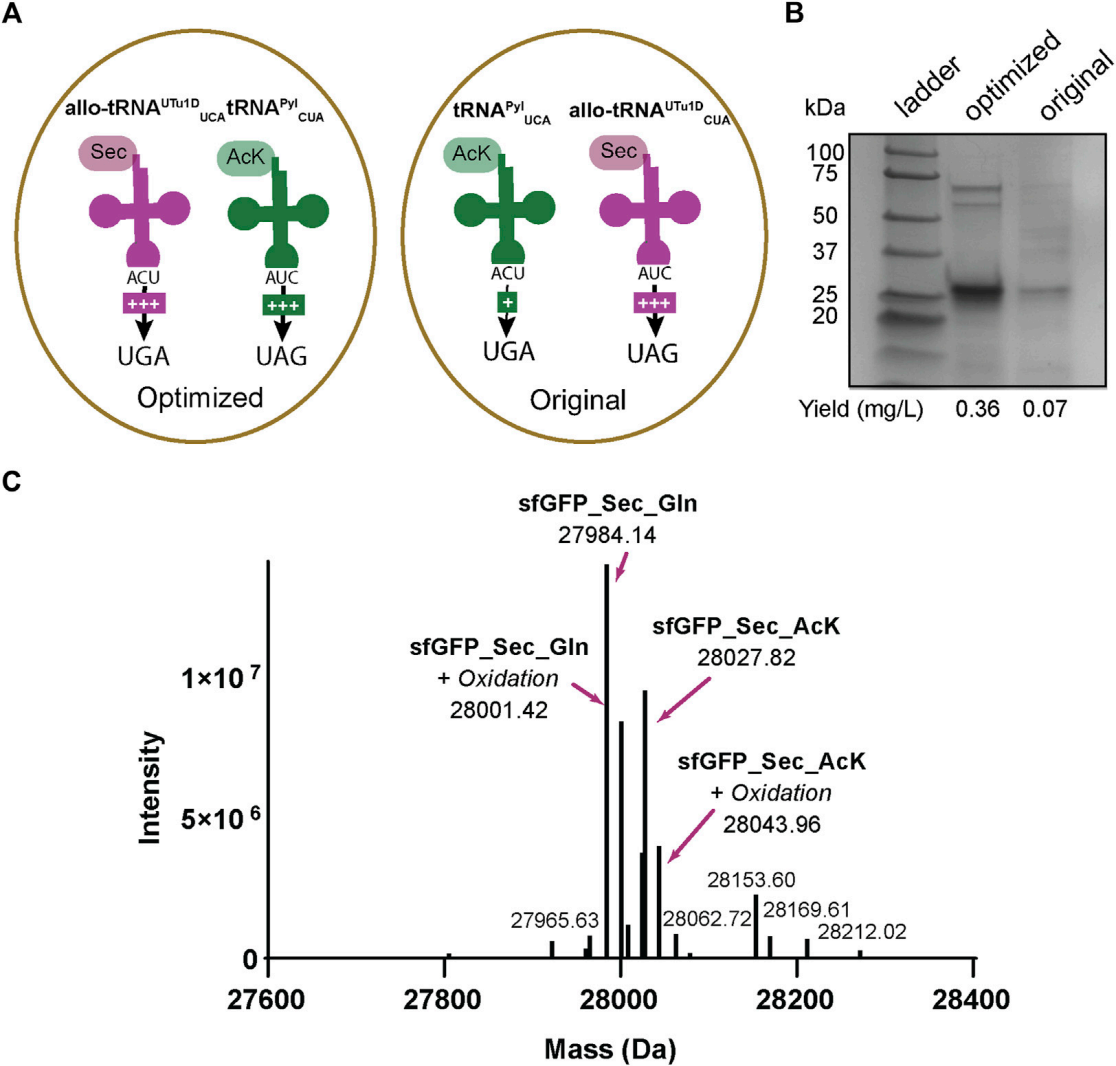
Production of AcK-seleno-sfGFP. **(A)** Schematic of tRNAs used and the respective codons they are expected to recode. Optimized conditions involved Sec insertion at position 2UGA with allo-tRNA^UTu1D^_UCA_ and *N*_ε_-acetyl-l-lysine (AcK) at 151UAG with tRNA^Pyl^_CUA_. Original conditions involved AcK insertion at position 2UGA with tRNA^Pyl^_UCA_ and Sec insertion at 151UAG with allo-tRNA^UTu1D^_CUA_. An increase in the amount of + signs correspond to the increased suppression observed. **(B)** SDS-PAGE shows a strong band around the 25 kDa marker, corresponding to the AcK-seleno-sfGFP size of 28 kDa. An increase in protein yield was found for the anticodon-optimized conditions compared to the original. **(C)** Intact MS data has multiple peaks which can be assigned to AcK-seleno-sfGFP (37%) and Gln-seleno-sfGFP (63%) with or without an oxidation state.

The sfGFP readthrough and intein assays demonstrated that AcK and Sec could be inserted into a single protein using two different stop codons. Therefore, we applied our integrated plasmid setup and growth conditions to express and purify acetylated seleno-sfGFP from *E. coli*. The location of the His_6_-tag on the C-terminus allowed the separation of full-length protein from truncated versions due to early termination (Figure 5A). This strategy produced a robust band at roughly 25 kDa correlating to our expected protein size (28 kDa) (Figure 6). We also compared our optimized system, which involves recoding UGA with Sec and UAG with AcK, to our original system (recoding UGA with AcK and UAG with Sec) (Figure 6A). In both systems, we obtained pure protein; however, our yields were increased roughly 5-fold with our optimized codon set compared to the original (Figure 6B). Mass spectrometry analysis of the optimized codon set confirmed the presence of acetylated seleno-sfGFP. Protein mass was also detected that corresponded to Gln instead of AcK at a proportion of 67%, suggesting that near-cognate suppression by tRNA^Gln^ can impact the final protein purity (Figure 6C). This was further verified with MS/MS data to show that indeed AcK or Gln was inserted at position 151 while Sec was always observed at position 2 (Supplementary Figure S2).

### 3.4 Recombinant purification of acetylated human selenoprotein GPx1

**FIGURE 7:**
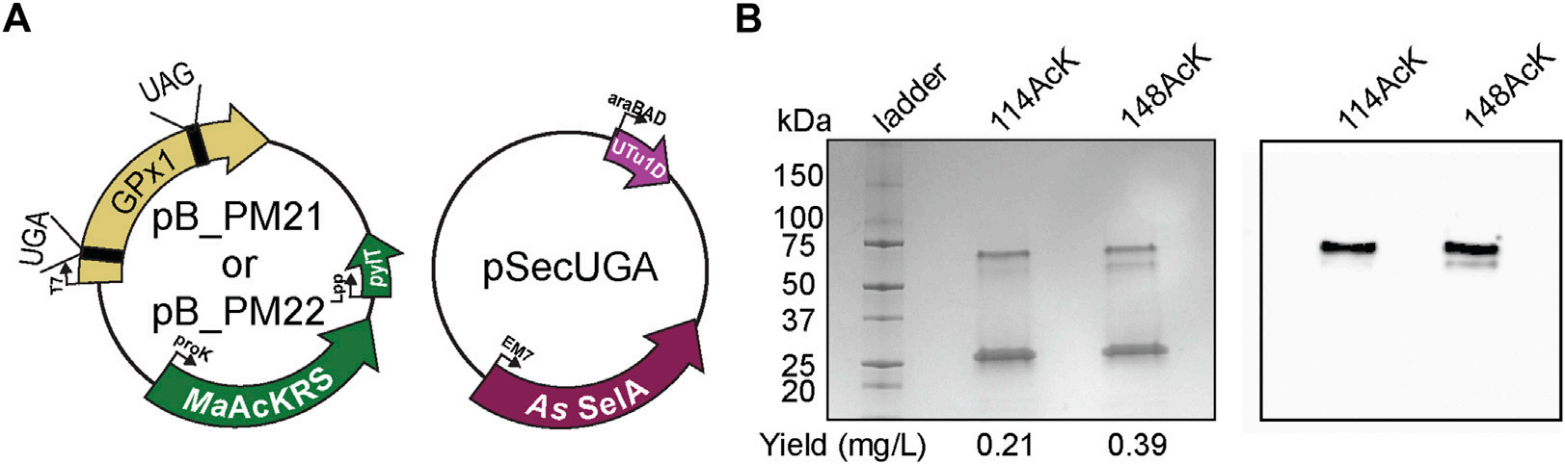
Production of post-translationally modified human GPx1. **(A)** Plasmids used for insertion of selenocysteine and *N*_ε_-acetyl-l-lysine (AcK) into GPx1 at UGA and UAG, respectively. Two variants were made with AcK at either position 114 or 148. **(B)** 4%–20% PAGE shows three bands on the gel for each variant, with the strongest one at the 25-kDa marker corresponding to monomeric GPx1 (24 kDa). An increase in protein yield was observed for 148AcK. Western blot with anti-AcK antibody targeted the two higher molecular weight bands.

To prove the versatility and utility of our system, we produced an authentic selenoprotein, human GPx1. GPx1 is predicted to be acetylated at different Lys residues based on murine GPx1 MS studies (Rardin et al., 2013). We individually targeted K114 and K148 together with the Sec at position 49. The protein was expressed under the same conditions with the same plasmid backbone as described for sfGFP (Figure 7A). Three bands were visible on the polyacrylamide gel, with the lower band corresponding to the expected size of monomeric GPx1 (25 kDa). In terms of protein yield, we found that GPx1_148AcK had roughly twice as much protein as GPx1_114AcK. This may be a result of codon context or the closer proximity of position 114 to 49Sec (Figure 7B). To confirm incorporation of AcK, a western blot with an anti-AcK antibody was used (Figure 7B). The western blot revealed the presence of acetylation in the higher molecular weight band which is approximately 30%–35% of the total protein (Supplementary Figure S3). We surmise that the lower molecular weight band corresponds to a GPx1 variant containing Gln (65%–70%) instead of AcK as suggested by the sfGFP MS data. This provides evidence that imply the potential role of AcK in GPx1 oligomerization, and emphasizes the necessity for a system which can study post-translationally modified selenoproteins.

## 4 Conclusion

Here we established a method for the simultaneous incorporation of selenocysteine and the ncAA *N*_ε_-acetyl-l-lysine into a protein at two targeted positions in *E*. *coli*. This method integrates pSec_Evol and MaPylRS:tRNA^Pyl^ non-sense translation systems for the first time (Figure 1). This was accomplished by characterizing the decoding efficiency, specificity, and compatibility of these systems. We found that allo-tRNA^UTu1D^ and *Ma*AcLysRS3:tRNA^Pyl^ are compatible when UGA and UAG are assigned to Sec and AcK, respectively (Figures 3B, C). In contrast, allo-tRNA^UTu1D^_CUA_ and tRNA^Pyl^_UCA_ lacked decoding fidelity as they translated both UGA and UAG codons, albeit with different efficiencies (Figures 2B, C). Thus, our results indicate that the codon specificity of these two translation systems should be an essential consideration when developing GCE applications, particularly when combining them with other systems. Another important consideration is the position of the non-sense codons within each protein as mRNA context can influence the suppressor tRNAs as well as the proximity of the two ncAAs. The off-target activity of these tRNAs with non-specific anticodons may hinder the accurate incorporation of the desired amino acid and drastically decrease protein yields by impacting the fitness of the host organism. Using this knowledge, we demonstrated that pSecUGA and *Ma*AcLysRS3:tRNA^Pyl^_CUA_ systems could be combined in *E*. *coli* to create a platform for synthesizing proteins containing these two important amino acids. This provides the groundwork to produce acetylated natural or synthetic selenoproteins. The utility of our method can be expanded by combining the pSecUGA system with other existing GCE platforms to facilitate the synthesis of selenoproteins containing other important PTMs or ncAAs with reactive side chains to expand protein functionalization (Young and Schultz, 2018; Shandell et al., 2021). Ultimately, this technology can enable the investigation of PTMs in naturally occurring selenoproteins and the development of artificial proteins endowed with unique chemical properties.

## Data Availability

The raw data supporting the conclusion of this article will be made available by the authors, without undue reservation.
