# Supplementary material for: Dual incorporation of non-canonical amino acids enables production of post-translationally modified selenoproteins
Source: Front Mol Biosci. 2023 Jan 24;10:1096261. doi: 10.3389/fmolb.2023.1096261 (PMC9902344; doi:10.3389/fmolb.2023.1096261)
Supplement: Supplementary file 1 [file DataSheet1.PDF]

Supplementary Material

1 Supplementary Figures and Tables

1.1 Supplementary Figures

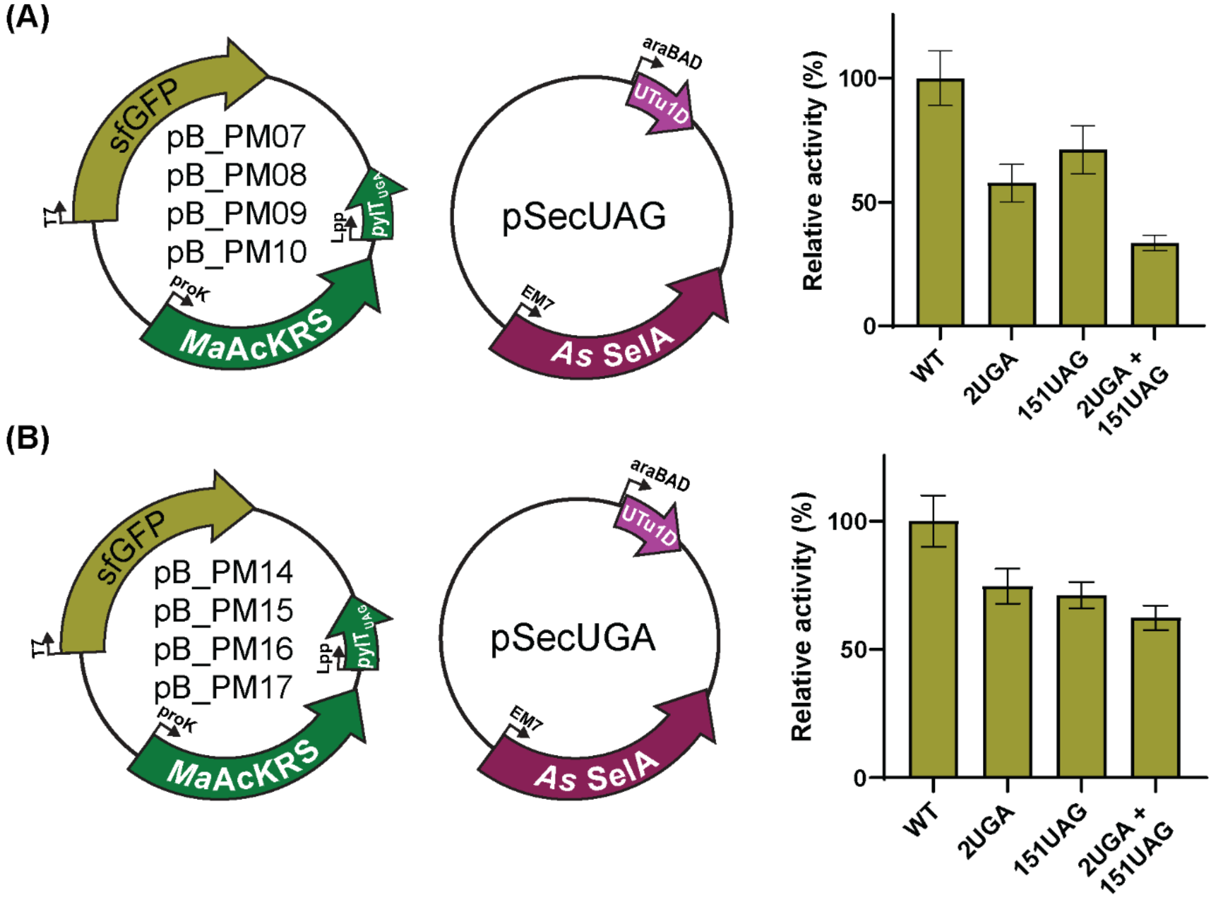

Supplementary Figure 1. Determining suppression efficiency for N_{ε}-acetyl-L-lysine (AcK) and selenocysteine (Sec) insertion. (**A**) Plasmids for AcK insertion at UGA and Sec at UAG to test efficiency of codon suppression individually (2UGA or 151UAG) or combined (2UGA + 151UAG) compared to wild-type (WT) sfGFP. Individual stop codon suppression was found to be 60 – 70% that of WT sfGFP while dual suppression dropped down to 34%. (**B**) Plasmids for AcK insertion at UAG and Sec at UGA to test efficiency of codon suppression individually (2UGA or 151UAG) or combined (2UGA + 151UAG) compared to wild-type (WT) sfGFP. Individual stop codon suppression was found to be 70 – 75% that of WT sfGFP while dual suppression dropped down to 62%. Data shown is the average of four biological replicates with error bars showing the standard deviation.

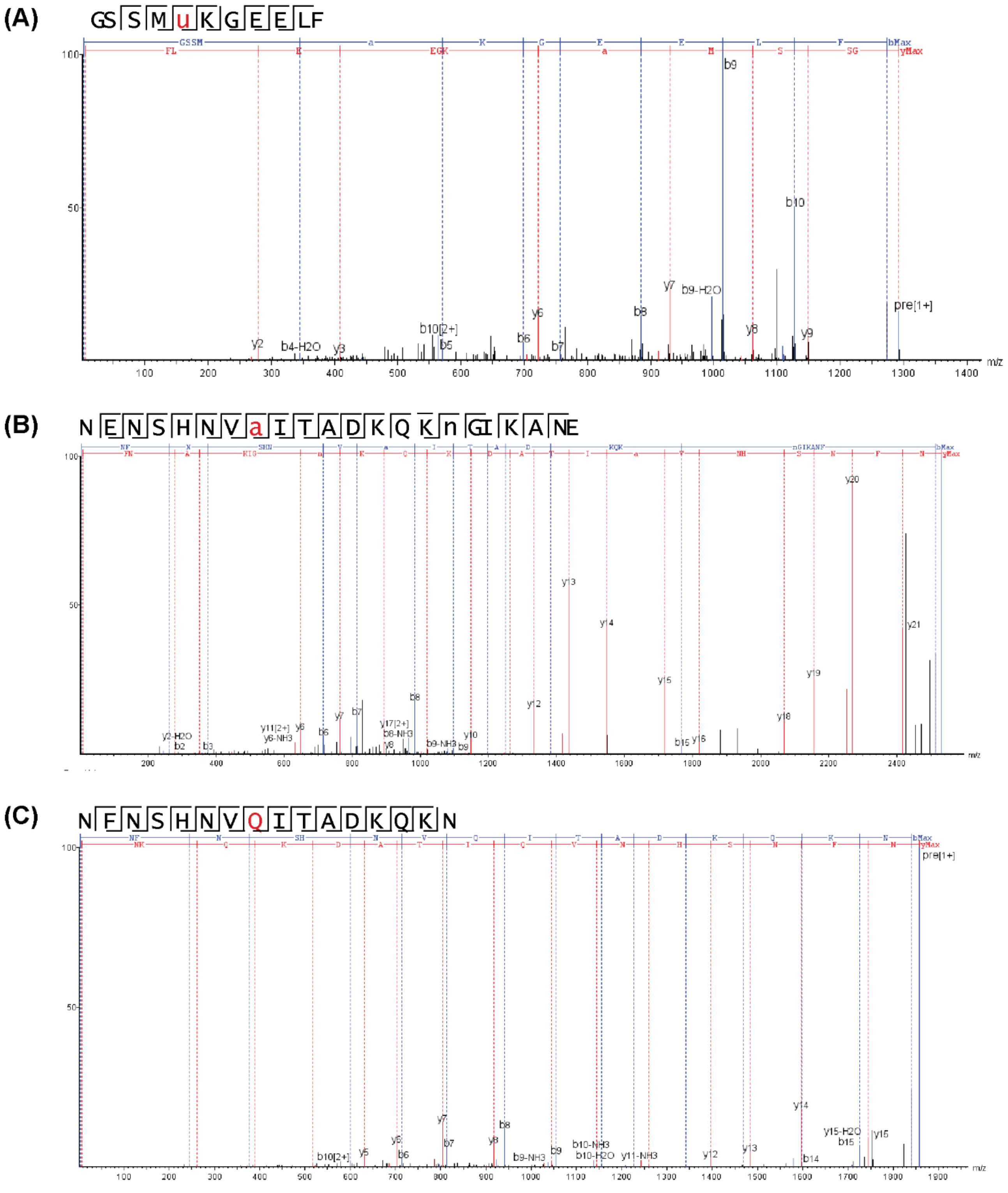

Supplementary Figure 2. Tandem mass spectrometry spectra for the most abundant peaks of AcK-seleno-sfGFP. (**A**) N-terminal peptide with selenocysteine (denoted with red u), (**B)** internal peptide with N_ε-acetyl-L-lysine (denoted with red a), (**C**) internal peptide with glutamine (denoted with a red Q).

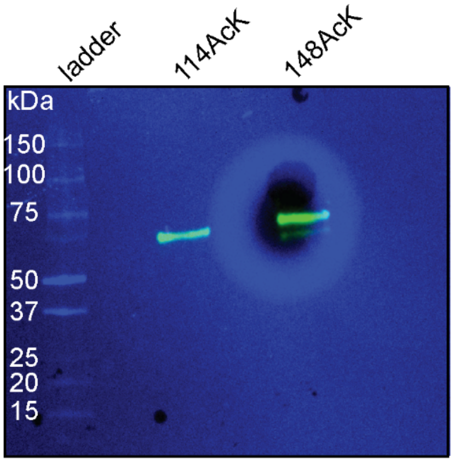

Supplementary Figure 3. Western blot overlay for post-translationally modified GPx1. Multichannel image of western blot shows the protein ladder (detected colorimetrically) and the acetylated protein band (detected with chemiluminescence).

1.2 Supplementary Tables

Table S1. Oligonucleotides used in this study

Name	Sequence (5’ → 3’)
**PM01**	CATCATCATTAAGCTGAGTTGGCTGCTG
**PM02**	CCAACTCAGCTTAATGATGATGATGATGATGTGAG
**PM03**	CAGCAGCATGTGAAAGGGCGAAGAGTTATTCA
**PM04**	CTTCGCCCTTTCACATGCTGCTGCCCATG
**PM05**	CACAACGTATAGATTACAGCTGACAAAC
**PM06**	CAGCTGTAATCTATACGTTGTGAGAG
**PM07**	TTTTTTCGGACGAGCAACTCGTCCGAAAAGCTTAATTAATTGATCTTGGCACC
**PM08**	AGGCATTTTGCTATTAAGGGATTGACGAGGGCGTATCTGCGCAGTAAGATGCGCCCCGCATTT CACACAGGAAACAC
**PM09**	CAATCCCTTAATAGCAAAATGCCTGAGGCAGCAGATCAATTC
**PM10**	CGGACGAGTTGCTCGTCCGAAAAAAGCGCGTTTCGGTGATG
**PM11**	GATCGTTCGCTCAAAGAAGCGGCGCCATCCGGGAGCTGCATG
**PM12**	GCGAAAGCTAAGGATTTTTTTAAGCTTGACGGTCACAGC
**PM13**	AAGCTTAAAAAAAATCCTTAGCTTTCGC
**PM14**	TGGCGCCGCTTCTTTG
**PM15**	CCGCTGGCTAGGTTTTAGAGACCCGC
**PM16**	GTCTCTAAAACCTAGCCAGCGGGGTTC
**PM17**	CAGCAGCATGTAGAAGGGCGAAGAGTTATTCA
**PM18**	CTTCGCCCTTCTACATGCTGCTGCCCATG
**PM19**	GTCCACTCAGTGAATCTCCGGAGATAGTTTG
**PM20**	CTCCGGAGATTCACTGAGTGGACAAGTAATG
**PM21**	GACGGGAACTTCAAATTCCTTGAAATGCCTCG
**PM22**	CAAGGAATTTGAAGTTCCCGTCTGCCACC
**PM23**	GAAGGAGATATACCATGGGCAGCAGCATGTGTGCTGCTCGG

**PM24**	GCCGCAAGCTTTTAGTG
**PM25**	GCTGCTGCCCATGGTATATC
**PM26**	CACCACCACTAAAAGCTTGCGGCGCTGAGTTGGCTGCTG
**PM27**	GCGTCCCTCTGAGGCACCACGGTCCGG
**PM28**	CCGTGGTGCCTCAGAGGGACGCCACATTC
**PM29**	GCTCTTCGAGTAGTGCGAGGTGAACG
**PM30**	CACCTCGCACTACTCGAAGAGCATGAAG
**PM31**	GACCGACCCCTAGCTCATCACCTGG
**PM32**	GTGATGAGCTAGGGGTCGGTCATAAG

Table S2. Plasmids used in this study

Plasmid name	Resist	Origin	ORF1		ORF2		ORF3		ORF4	
			Prom	Gene	Prom	Gene	Prom	Gene	Prom	Genes
pSecUAG	Spec	pRSF	P_{BAD}	allotRNA$^{UTu1D}_{CUA}$	P_{SufS}	*E.coli* SufS[C364A]	P_{EM7}	*As* SelA [Evol]	P_{SelD}	*As* SelD *Td* Trx
pSecUGA	Spec	pRSF	P_{BAD}	allotRNA$^{UTu1D}_{UCA}$	P_{SufS}	*E.coli* SufS[C364A]	P_{EM7}	*As* SelA [Evol]	P_{SelD}	*As* SelD *Td* Trx
pB_sfGFP	Amp	pBR322	P_{T7}	sfGFP	-	-	-	-	-	-
pB_PM01	Amp	pBR322	P_{T7}	sfGFP[TAA]	-	-	-	-	-	-
pB_PM02	Amp	pBR322	P_{T7}	sfGFP[2TGA_TAA]	-	-	-	-	-	-
pB_PM03	Amp	pBR322	P_{T7}	sfGFP[151TAG_TAA]	-	-	-	-	-	-
pB_PM04	Amp	pBR322	P_{T7}	sfGFP[TAA]	P_{proK}	*Ma*AcLysRS3-IP	-	-	-	-
pB_PM05	Amp	pBR322	P_{T7}	sfGFP[2TGA_TAA]	P_{proK}	*Ma*AcLysRS3-IP	-	-	-	-
pB_PM06	Amp	pBR322	P_{T7}	sfGFP[151TAG_TAA]	P_{proK}	*Ma*AcLysRS3-IP	-	-	-	-
pB_PM07	Amp	pBR322	P_{T7}	sfGFP[TAA]	P_{proK}	*Ma*AcLysRS3-IP	P_{Lpp}	tRNA$^{Pyl}_{UCA}$	-	-
pB_PM08	Amp	pBR322	P_{T7}	sfGFP[2TGA_TAA]	P_{proK}	*Ma*AcLysRS3-IP	P_{Lpp}	tRNA$^{Pyl}_{UCA}$	-	-
pB_PM09	Amp	pBR322	P_{T7}	sfGFP[151TAG_TAA]	P_{proK}	*Ma*AcLysRS3-IP	P_{Lpp}	tRNA$^{Pyl}_{UCA}$	-	-
pB_PM10	Amp	pBR322	P_{T7}	sfGFP[2TGA_151TAG _TAA]	P_{proK}	*Ma*AcLysRS3-IP	P_{Lpp}	tRNA$^{Pyl}_{UCA}$	-	-
pB_04	Amp	pBR322	P_{T7}	sfGFP[204TAG_M86]	-	-	-	-	-	-
pB_PM11	Amp	pBR322	P_{T7}	sfGFP[204TAG_M86 _TAA]	-	-	-	-	-	-
pB_PM12	Amp	pBR322	P_{T7}	sfGFP[2TGA_ 204TAG_M86_TAA]	-	-	-	-	-	-
pB_PM13	Amp	pBR322	P_{T7}	sfGFP[2TGA_ 204TAG_M86_TAA]	P_{proK}	*Ma*AcLysRS3-IP	P_{Lpp}	tRNA$^{Pyl}_{UCA}$	-	-
pB_PM14	Amp	pBR322	P_{T7}	sfGFP[TAA]	P_{proK}	*Ma*AcLysRS3-IP	P_{Lpp}	tRNA$^{Pyl}_{CUA}$	-	-
pB_PM15	Amp	pBR322	P_{T7}	sfGFP[2TGA_TAA]	P_{proK}	*Ma*AcLysRS3-IP	P_{Lpp}	tRNA$^{Pyl}_{CUA}$	-	-
pB_PM16	Amp	pBR322	P_{T7}	sfGFP[151TAG_TAA]	P_{proK}	*Ma*AcLysRS3-IP	P_{Lpp}	tRNA$^{Pyl}_{CUA}$	-	-
pB_PM17	Amp	pBR322	P_{T7}	sfGFP[2TGA_151TAG _TAA]	P_{proK}	*Ma*AcLysRS3-IP	P_{Lpp}	tRNA$^{Pyl}_{CUA}$	-	-

pB_PM18	Amp	pBR322	P_{T7}	sfGFP[2TAG_ 204TGA_M86_TAA]	P_{proK}	*Ma*AcLysRS3-IP	P_{Lpp}	$tRNA^{Pyl}_{CUA}$	-	-
pB_PM19	Amp	pBR322	P_{T7}	GPx1[49TAG]	P_{proK}	*Ma*AcLysRS3-IP	P_{Lpp}	$tRNA^{Pyl}_{CUA}$	-	-
pB_PM20	Amp	pBR322	P_{T7}	GPx1[49TGA]	P_{proK}	*Ma*AcLysRS3-IP	P_{Lpp}	$tRNA^{Pyl}_{CUA}$	-	-
pB_PM21	Amp	pBR322	P_{T7}	GPx1[49TGA_114TAG]	P_{proK}	*Ma*AcLysRS3-IP	P_{Lpp}	$tRNA^{Pyl}_{CUA}$	-	-
pB_PM22	Amp	pBR322	P_{T7}	GPx1[49TGA_148TAG]	P_{proK}	*Ma*AcLysRS3-IP	P_{Lpp}	$tRNA^{Pyl}_{CUA}$	-	-
